# The importance of the whole: Topological data analysis for the network neuroscientist

**DOI:** 10.1162/netn_a_00073

**Published:** 2019-07-01

**Authors:** Ann E. Sizemore, Jennifer E. Phillips-Cremins, Robert Ghrist, Danielle S. Bassett

**Affiliations:** Department of Bioengineering, School of Engineering and Applied Sciences, University of Pennsylvania, Philadelphia, USA; Department of Bioengineering, School of Engineering and Applied Sciences, University of Pennsylvania, Philadelphia, USA; Department of Mathematics, College of Arts and Sciences, University of Pennsylvania, Philadelphia, USA; Department of Bioengineering, School of Engineering and Applied Sciences, University of Pennsylvania, Philadelphia, USA; Department of Physics & Astronomy, College of Arts and Sciences, University of Pennsylvania, Philadelphia, USA; Department of Electrical & Systems Engineering, School of Engineering and Applied Sciences, University of Pennsylvania, Philadelphia, USA; Department of Neurology, Perelman School of Medicine, University of Pennsylvania, Philadelphia, USA

**Keywords:** Topological data analysis, Applied topology, Persistent homology

## Abstract

Data analysis techniques from network science have fundamentally improved our understanding of neural systems and the complex behaviors that they support. Yet the restriction of network techniques to the study of pairwise interactions prevents us from taking into account intrinsic topological features such as cavities that may be crucial for system function. To detect and quantify these topological features, we must turn to algebro-topological methods that encode data as a simplicial complex built from sets of interacting nodes called simplices. We then use the relations between simplices to expose cavities within the complex, thereby summarizing its topological features. Here we provide an introduction to persistent homology, a fundamental method from applied topology that builds a global descriptor of system structure by chronicling the evolution of cavities as we move through a combinatorial object such as a weighted network. We detail the mathematics and perform demonstrative calculations on the mouse structural connectome, synapses in *C. elegans*, and genomic interaction data. Finally, we suggest avenues for future work and highlight new advances in mathematics ready for use in neural systems.

## Author Summary

For the network neuroscientist, this exposition aims to communicate both the mathematics and the advantages of using tools from applied topology for the study of neural systems. Using data from the mouse connectome, electrical and chemical synapses in *C. elegans*, and chromatin interaction data, we offer example computations and applications to further demonstrate the power of topological data analysis in neuroscience. Finally, we expose the reader to novel developments in applied topology and relate these developments to current questions and methodological difficulties in network neuroscience.

## INTRODUCTION

Network science now branches far into applied fields such as medicine (Barabási, Gulbahce, & Loscalzo, 2011), genetics (Alon, 2007), physics (Papadopoulos, Porter, Daniels, & Bassett, 2018), sociology (Wasserman, 1994), ecology (Proulx, Promislow, & Phillips, 2005), and neuroscience (Sporns, 2014). Such breadth is made possible by the discipline’s roots in the abstract field of graph theory. Using this deep theoretical foundation, we now analyze network models with an ease and finesse previously not possible, while confidently continuing to develop the underlying mathematical framework. For the most elementary application of graph-based methods, we only require our system to be composed of units and their pairwise relations, a simple requirement that is commonly met in the modeling of many biological systems, including the brain. Yet often still another level of organization exists in which groups of units all serve a function together (perhaps via a similar process or capacity), implying that any subset of actors in a group also has this relation. Many studies spanning cellular and areal scales stress the importance of such higher order interactions (Bassett, Wymbs, Porter, Mucha, & Grafton, 2014; Ganmor, Segev, & Schneidman, 2011). Algebraic topology presents us with a language with which to encode, study, and manipulate such structures with higher order relations. In addition, the field of algebraic topology gifts us with nearly 100 years of theoretical groundwork distinct from and yet complementary to that offered by graph theory.

**Figure 1.**
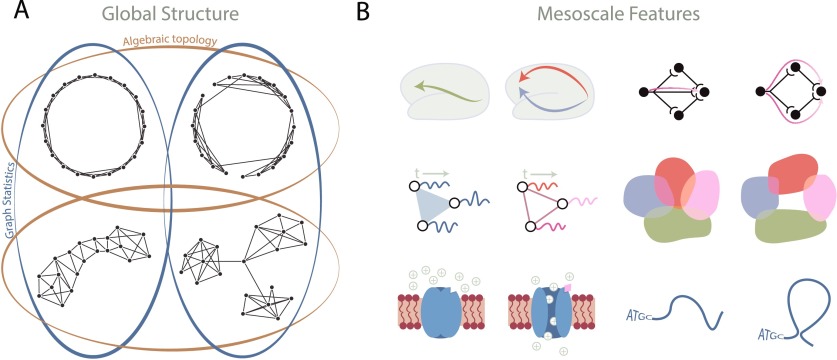
Topology at the global and mesoscales. (A) An illustration of four graphs that can be separated into two different sorts of groups based on either algebraic topology or graph statistics. Algebraic topology (gold) sees the circular nature of the top two graphs as distinct from the linear or star-like global structures in the bottom two graphs. In contrast, graph statistics (blue) see the graphs on the left side as being similar, because they have the same degree distribution and no modular structure; graph statistics also see the graphs on the right side as being similar because they both have three modules and share the same degree distribution. (B) Examples of topology in neural systems at different scales. (Left, top) Topologically distinct paths serve to either directly communicate or parallel process information at the macroscale. (Left, middle) Three oscillators whose dynamics are correlated in an all-to-all fashion versus in a pairwise-only fashion differ in topology. (Left, bottom) A ligand-gated channel that is closed (topologically trivial) versus open (topologically nontrivial) to allow the flow of ions. (Right, top) Activity flow in neurons may follow a linear or a cavity-surrounding, information-reinforcing trajectory. (Right, middle) Neural place fields covering the entire space versus leaving gaps, thus differing in topology. (Right, bottom) Linear DNA versus a long-range 3D interaction that creates a topologically nontrivial feature.

Algebraic topology generally concerns itself with the “shape” of topological spaces, or—more precisely—those properties of a space that remain invariant under stretching and shrinking (note: for a more formal treatment of the subject, the interested reader is advised to consult (Ghrist, 2014; Hatcher, 2002; Munkres, 2000)). Focusing on these invariants provides a perspective that is fundamentally distinct from that offered by more geometrically informed measures, generally termed “network topology.” As an example, Figure 1 shows four graphs that can be thought of as topological spaces; the top row contains globally circular graphs and the bottom row contains globally noncircular graphs. Algebraic topology sees (i) that the graphs in the top row are similar because they are organized into one circular loop that surrounds a “hole,” and (ii) that the graphs in the bottom row are similar because they are not organized into such a global “hole”-enclosing loop.

Algebraic topology has been usefully applied at macro- and microscales to elucidate the function of neural systems. For example, both place fields and neural codes contain and are driven by fundamentally topological notions (Curto, 2017; Curto & Itskov, 2008). Topological data analysis has also been used in conjunction with a model of spiking neurons in rat hippocampus during a simulated trajectory through space to suggest optimal parameters for learning the mapped space (Dabaghian, Mémoli, Frank, & Carlsson, 2012) as well as finding that the synchronization of theta phases and gamma waves in a computational model allow for learning topological information in the environment (Basso, Arai, & Dabaghian, 2016). DNA processes such as recombination can be modeled using topology, and furthermore, the resulting topology indicates the number of steps taken in the given process (Jonoska & Saito, 2004). Additionally, Arai, Brandt, and Dabaghian (2014) used topological methods to test the influence of *θ* band precession on spatial learning. Furthermore, Giusti, Pastalkova, Curto, and Itskov (2015) employed these methods to determine whether correlations of recorded pyramidal neuronal activity in the rat hippocampus contain a natural geometric structure. At the macroscale, persistent homology has been used to detect cyclic motifs thought to form paths of parallel information processing in the structural human brain network (A. E. Sizemore et al., 2017). In networks constructed from functional neuroimaging data, tools to identify cycles have been used to study changes in neurophysiology accompanying the learning of new visuo-motor skills (B. Stolz, 2014) and to distinguish the effects of drug versus placebo on neural dynamics (Petri et al., 2014). Finally, topological simplification methods such as Mapper (Singh, Mémoli, & Carlsson, 2007) have allowed for visualization of brain trajectories throughout task switching (Saggar et al., 2018) and for the identification of a new breast cancer subtype (Nicolau, Levine, & Carlsson, 2011; for an overview see Patania, Vaccarino, & Petri, 2017).

The consideration of topology in data analysis is relatively new (Carlsson, 2009), although its methods are quite appropriate given their freedom from coordinates and robustness to noise. As with any new application of pure mathematics, mathematicians are continually pushing the technology forward, and biologists are finding new questions that can now be addressed with these tools. However, since the formal mathematics underpinning topology is likely to be relatively unfamiliar to many neuroscientists, a nontrivial start-up cost may be required in order to accurately and fruitfully apply the tools to any particular neural system. Here we provide an exposition that aims to lower this activation energy by offering a basic introduction to the primary mathematics behind a main tool in topological data analysis, and examples of previous and possible applications of the tool in neuroscience.

Specifically, in this paper we will take the reader through an introduction to the mathematics of persistent homology and the analyses that are made possible by this mathematics. We will restrict the mathematical definitions to the particular case of basic persistent homology applications. To demonstrate concepts, we will perform the related analyses on three datasets covering multiple species and multiple spatial scales of inquiry: the mouse connectome (Oh et al., 2014; Rubinov, Ypma, Watson, & Bullmore, 2015), electrical and chemical synapses in *C. elegans* (Varshney, Chen, Paniagua, Hall, & Chklovskii, 2011), and genomic interaction data collected from Hi-C experiments (Van Berkum et al., 2010). Finally, we will discuss other potential applications, highlighting new questions answerable with up-and-coming topological methods. For the interested reader, we emphasize that more detailed reviews and tutorials are available (Edelsbrunner & Harer, 2008; Ghrist, 2017), as well as an overview of available software packages (Otter, Porter, Tillmann, Grindrod, & Harrington, 2017).

## WHEN SHOULD WE USE TOPOLOGICAL DATA ANALYSIS?

Before delving into the mathematics, it is important to first pause and ask if the system of interest is well suited for these methods and how topology might be interpreted in this system. We suggest two specific considerations when evaluating the application of topological data analysis to a specific scientific enquiry. First, are higher order interactions (more than pairwise) important in the system? Examples of such higher order interactions are evident across spatial scales in neuroscience: groups of brain regions may serve a similar function, proteins may combine into complexes, or neurons may operate in groups. For such systems, one can apply topological data analysis methods to obtain a picture of the global topology, which in turn can more comprehensively reveal the system’s intrinsic structure (Giusti et al., 2015) and distinguish that structure from noise (Horak, Maletić, & Rajković, 2009; M. Kahle, 2014; Petri, Scolamiero, Donato, & Vaccarino, 2013; A. Sizemore, Giusti, & Bassett, 2017).

Second, what does a topological cavity mean in the system under study and why might such a feature be important? This can sometimes be difficult to imagine initially, so we provide examples of how differing topology in neural systems may serve unique functions in Figure 1B. Perhaps the system is the structural connectome, where a cavity in diffusion MRI could indicate axonal dropout (Demiralp, 2013). In networks constructed from functional imaging data, a cavity might arise when multiple small groups of regions display correlated activity, but when the regional activity profiles are not all together similar. In chromatin, previous studies have shown that long-range interactions regulate gene expression (Lieberman-Aiden et al., 2009; Sanyal, Lajoie, Jain, & Dekker, 2012), and disruption to or changes in these interactions might play a role in disease (Ahmadiyeh et al., 2010; Kleinjan & van Heyningen, 2005). Although we have included the above and those in Figure 1B as examples, we note that creativity continues to fuel many more interpretations than can be expressed here.

## PIECES AND PARTS OF THE SIMPLICIAL COMPLEX

**Figure 2.**
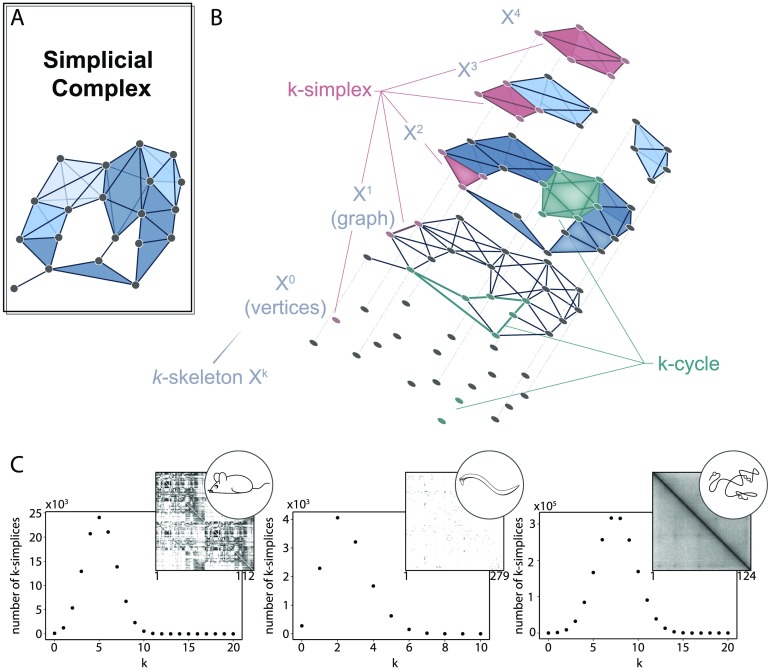
The simplicial complex. (A) A simplicial complex with simplicies colored by dimension. (B) Peering into the simplicial complex by separating it into the *k*-skeleta. An example *k*-simplex is colored in burgundy, an example *k*-chain is colored in salmon, and several example *k*-cycles are colored in teal for each dimension. (C) The distribution of *k*-simplices in the clique complex created from the (*left*) mouse interareal connectome, (*middle*) *C. elegans* electrical and chemical synapses, and (*right*) genomic interaction Hi-C data indicating distances between points on the linear genome. The adjacency matrix associated with each system is displayed in the upper right corner of each plot.

In network science, we might commonly translate our data into a binary graph and then proceed with techniques anchored in graph theory. For topological data analysis, we instead translate our data into an object called a *simplicial complex* (Figure 2A), which allows us to draw from an expanded pool of topological methods. Instead of exclusively recording the presence or absence of pairwise relations, here relational objects can connect any number of nodes. Such a unit of *k* + 1 nodes is called a *k*-simplex (Figure 2B, salmon), which is geometrically the convex hull of its *k* + 1 affinely positioned vertices: one node is a 0-simplex, an edge is a 1-simplex, a filled triangle is a 2-simplex, and so on. We write a *k*-simplex formed with nodes *v*_0_, …, *v*_*k*_ as {*v*_0_, …, *v*_*k*_}. A collection of nicely constructed simplices forms a simplicial complex: a set of nodes *V* and a collection *K* of simplices subject to the rule that if *s* is a simplex in *K* and *s*′ ⊆ *s*, then *s*′ ∈ *K*. This rule prevents having a 2-simplex in the complex without containing its constituent edges (1-simplices) or vertices (0-simplices), and similar atrocities. Finally, it may be convenient to reference particular layers or dimensions of the simplicial complex. Formally this is the *k*-skeleton, denoted *X*^*k*^, or the collection of all simplices with dimension at most *k* in the simplicial complex (Figure 2B, gray). Looped patterns of *k*-simplices called *k*-*cycles* (Figure 2B, green) are of particular interest within the complex and will be more formally defined in the coming sections.

After constructing a simplicial complex (see Box 1), we can obtain a first glimpse of the system’s structure by calculating the simplex distribution (Figure 2C). The number of simplices in each *k*-skeleton can provide insight into how the system functions (Reimann et al., 2017) by reflecting the size of functional units, or it can help distinguish structure from noise (A. Sizemore et al., 2017). In a graph reflecting closeness of points in space, one expects that if two edges connect three nodes, the third edge that closes the triangle is likely to exist. As a higher-dimensional analog, if we construct a simplicial complex from data that has geometric constraints such as in the genomic interaction data (two nodes closer along the linear DNA are more likely to be physically near each other), we will see many larger simplices. One can even generalize many network measures, including centrality, to simplicial complexes in order to more thoroughly describe and quantify the system’s structure (Estrada & Ross, 2017).

**Box 1.**  **From data to simplicial complex**

**Figure 3.**
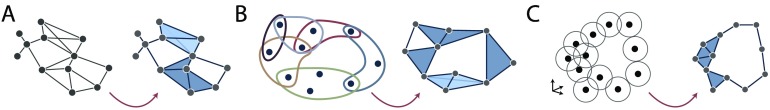
Simplicial complexes from data. (Left) Clique complex created from a binary graph. (Middle) Nerve complex from overlapping groups of nodes. (Right) Vietoris-Rips complex from a point cloud.

There are multiple ways to encode data into a simplicial complex. If the data is already in the form of a binary graph, the most basic representation is called the *clique complex*. The clique complex (or flag complex) is the simplicial complex formed from assigning a *k*-simplex to each (*k* + 1)-clique in the graph (see Figure 3A). This representation is generally favored when no further information beyond pairwise relations is known, since it requires the fewest choices in the designation of simplices.

A second interesting way to create a complex from data can be used when higher order relations are known, such as groups of nodes sharing a feature. In this case, one can create the nerve complex that is a *#simplices* × *#vertices* matrix recording the vertices that form each simplex (corresponding to a feature) in the complex. Note that we assume downward completion of simplices; that is, if {*v*_*i*_, *v*_*j*_, *v*_*k*_} is a simplex, then we include {*v*_*i*_, *v*_*j*_}, {*v*_*j*_, *v*_*k*_}, {*v*_*i*_, *v*_*k*_}, and so on, as simplices as well. This representation is particularly suitable for data that comes in the form of nodes and groups, since in this object, one could have three edges connect in a triangle without filling in the encompassed area (i.e., we could have three 1-simplices arranged in a triangle but are not part of a 2-simplex, see Figure 3B). An example context in which it may be fruitful to use the nerve complex is when studying correlation among system units: here, we could imagine three vertices whose activity profiles are correlated in pairs, but not correlated all together (see Figure 1B). See (Giusti, Ghrist, & Bassett, 2016) for details on this example and for a few additional complexes that can be used for data encoding.

If the data instead comes as a point cloud, such as points in the brain’s state space, we can build a simplicial complex by choosing some value *ϵ* and drawing in a *k*-simplex for *k* + 1 nodes that are a pairwise distance < *ϵ* apart. This representation is called the Vietoris-Rips complex (see Figure 3C) and is used to infer the shape of data (Carlsson, 2009). As an extension, if points have an associated size or other feature (e.g., the van der Waals radius for points representing atoms in a protein crystal structure, Gameiro et al., 2015), then one can create the alpha complex where the parameter *ϵ* is weighted based on the point size (Edelsbrunner, 1995).

**Figure 4.**
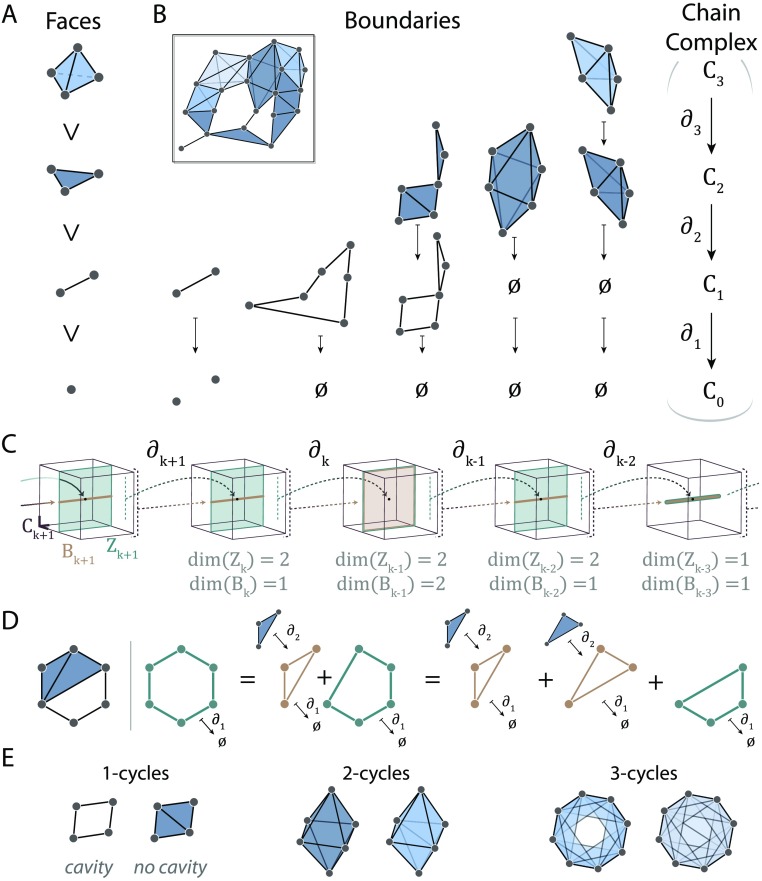
Chain complexes and homology. (A) A 3-simplex (top) has 2-simplices, 1-simplices, and 0-simplices as faces. (B) Boundary maps take *k*-chains to their boundaries. Examples shown for dimensions 1 through 3. These boundary maps connect the chain groups forming the chain complex (right). (C) Depiction of a chain complex with 3-dimensional chain groups (maroon boxes). Boundaries (gold) and cycles (green) are defined using the boundary operator and may span the same space, or the boundary space may be a strict subspace of the cycle space. (D) Given the simplicial complex shown at the (left), the three green cycles are equivalent because they differ by boundary cycles (gold). (E) Examples of *k*-cycles for *k* = 1 (left), *k* = 2 (middle), and *k* = 3 (right). For each *k* we show a non-trivial (cavity-enclosing) cycle on the left and a trivial cycle on the right.

We now turn to a description of how we will leverage all of this higher order information within the simplicial complex to reveal a system’s essential topological features. We begin with the simple observation that simplices contain other smaller simplices and that this containment provides a notion of relation among simplices and, furthermore, provides additional structural information. More specifically, a simplex in a simplicial complex is maximal if it is not contained in any larger simplex. Furthermore, any subset of a simplex is called a face (Figure 4A). For example, if {*v*_*i*_, *v*_*j*_, *v*_*k*_} is a 2-simplex, it contains a 1-simplex {*v*_*i*_, *v*_*j*_}, where {*v*_*i*_, *v*_*j*_} is a face of {*v*_*i*_, *v*_*j*_, *v*_*k*_}. We can easily keep track of facial relations within a simplicial complex which can be used to highlight important properties, such as which *k*-simplices are maximal and which are faces of higher dimensional simplices.

### Chain Groups and Boundaries

We imagine cavities within simplicial complexes as akin to bubbles under water. Now a bubble must first and almost trivially be void of water, and secondly must be encapsulated by a water shell (the bubble’s surface). Contrastingly, the air completely above the water is not in a water bubble, since it does not live in a shell of water. In the same way, cavities in simplicial complexes must be void of higher simplices as well as completely enveloped, or bounded, by simplices. As we will see below, this notion of a boundary is crucial to detecting such topological features.

Let’s begin with a familiar matrix that records node membership within edges. This is a *#nodes* × *#edges* binary matrix with a 1 in position *i*, *j* if the *i*^*th*^ node is involved in the *j*^*th*^ edge (in simplicial complex terms we would say 0-simplex {*v*_*i*_} is a face of 1-simplex {*v*_*i*_, *v*_*j*_}). We will call this matrix ∂_1_. For simplicity in computations, we use vector spaces with binary coefficients so that all vectors record “0” or “1” in slots, and vector addition proceeds via binary arithmetic: edges and vertices are either “off“ or “on.” Recall that any matrix is a linear map between vector spaces. Here we are mapping from the vector space formed by assigning one basis element per edge to the vector space formed by assigning one basis element per node. These vector spaces are called the first and zeroth chain groups, *C*_1_ and *C*_0_, respectively, and their elements (vectors) are also called 1- or 0-chains.

This matrix houses a large amount of structural information. First, if we send a 1-chain corresponding to one edge through this map, it is sent to the sum of its two end nodes, or its boundary. Not surprisingly, ∂_1_ is called the boundary operator. Second, note that we can make 1-chains in *C*_1_ corresponding to paths in the simplicial complex. Then sending a 1-chain corresponding to a path through ∂_1_ will give its two boundary nodes since the internal nodes in the path will cancel because of the binary arithmetic. In particular, if the path is a cycle, then the beginning and end nodes are the same, so necessarily its boundary is null. Recalling that the kernel of a matrix is the subspace formed by all vectors sent to 0 by the matrix, one can show that the kernel of ∂_1_ is precisely the subspace spanned by these cycles. If the kernel of ∂_1_ has significance, what then of the image, ∂_1_(*C*_1_) ⊆ *C*_0_? In fact, the image consists of all 0-chains that coincide with the nodes at the *boundary* of edges and paths in the complex.

Now we again lift from networks to complexes. If we let *C*_*k*_ (the *k*^*th*^ chain group) denote the vector space with basis elements coinciding with *k*-simplices, then we can similarly create the boundary map ∂_*k*_ by creating a dim(*C*_*k*−1_) × dim(*C*_*k*_) binary matrix with entry *i*, *j* = 1 only if the *i*^*th*^ (*k* − 1)-simplex is a face of the *j*^*th*^ *k*-simplex. For example, the boundary of a 1-chain corresponding to a path is the sum of its beginning and end vertices, the boundary of a 2-chain corresponds to the sum of its surrounding edges, and the boundary of a 3-chain corresponds to a shell-forming sum of 2-simplices (see Figure 4B for examples). As in the lower dimension case, the boundary operator ∂_*k*_ has as a kernel *k*-cycles and sends *k*-chains to their boundaries, which in turn must be *k* − 1 cycles. Together, the chain groups *C*_*k*_ and boundary operators ∂_*k*_ : *C*_*k*_ → *C*_*k*−1_ form a chain complex (Figure 4B, right) in which the image of ∂_*k*_ in *C*_*k*−1_ (all (*k* − 1)-chains that are boundaries of *k*-chains) is contained within the kernel of ∂_*k*−1_. We think of the chain complex as an instruction manual for assembling the simplicial complex from a collection of simple building blocks: simplices.

To summarize: we can create matrices ∂_*k*_ sending the vector space of *k*-simplices to the vector space of (*k* − 1)-simplices that encode the structure of the simplicial complex, including its cycles and boundaries.
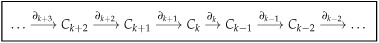


### Homological Algebra

Our goal is to find the topological features—cavities of different dimensions—of the simplicial complex. We are close: we now know how to find cycles of dimension *k*, namely *Z*_*k*_ = ker ∂_*k*_ or the *k*-cycle subspace. If there are topological cavities in the simplicial complex, some of these cycles will surround them, but still other cycles will instead be boundaries of higher dimensional simplices. Those *k*-cycles that are boundaries of (*k* + 1)-chains form the *k*-boundary subspace *B*_*k*_, a subspace of *Z*_*k*_. Intuitively, if *Z*_*k*_ records all the *k*-cycles, *B*_*k*_ records which of these are “footprints” left by higher dimensional simplices. We could attempt to study each of the boundary maps ∂*k* : *C*_*k*_ → *C*_*k*−1_ in isolation, but homological algebra tells us that we will gain much more by considering the entire chain complex as a whole (a lesson relearned in persistent homology).

To appreciate this fact, consider the example chain complex shown in Figure 4C. Linking all of these matrices together, we can see how the information in one map might overlap with the information provided by the subsequent map. In this example, the image of ∂_*k*+1_ (i.e., *B*_*k*_) is a subspace of the two-dimensional ker(∂_*k*_) = *Z*_*k*_. So *B*_*k*_ ⊂ *Z*_*k*_, and there is one dimension of information in *Z*_*k*_ that *B*_*k*_ does not see. Next, we observe that *Z*_*k*−1_ = *B*_*k*−1_. Here all of the information in *Z*_*k*−1_ is also contained in *B*_*k*−1_. Cavities arise when we have cycles that are not also boundaries. Thus, we lastly need to understand how to extract the discrepancy between the information housed in *Z*_*k*_ and the information housed in *B*_*k*_.

As an example, we turn to dimension 1 in the simplicial complex shown in Figure 4D. Each green cycle surrounds the cavity within the simplicial complex, but they differ from one another by the addition of a boundary cycle (gold). This observation is indicative of an over arching rule: when we add boundary cycles *b* ∈ *B*_*k*_ to cycles ℓ ∈ *Z*_*k*_, the resulting cycle ℓ + *b* will surround the same cavity (or cavities, or no cavities) as ℓ. Then to extract the topological cavities, we desire the *equivalence classes* of cycles ℓ ∈ *Z*_*k*_ where two cycles ℓ_1_, ℓ_2_ ∈ *Z*_*k*_ are equivalent if ℓ_1_ = ℓ_2_ + *b* for some *b* ∈ *B*_*k*_. Recall that the equivalence class of an element *σ* is the set of all elements equivalent to *σ* and denoted [*σ*]. So any cycle ℓ ∈ [green cycle] will surround the cavity within this example simplicial complex.

The cycle space *Z*_*k*_ contains a large amount of information about the structure (Shanker, 2007), much more than we need for our purposes, in fact. Since adding boundary cycles to a given cycle does not change the cavities it surrounds, then all of the topological information we seek is stored in the cycle space that is not altered by movements along the dimensions within the boundary subspace. Formally compressing the cycle space in this way to get the equivalence classes of *k*-cycles is called taking the vector space quotient: here *Z*_*k*_/*B*_*k*_ =: *H*_*k*_ is called the *k*^th^ homology group of the simplicial complex. That is, any boundary cycle (*b* ∈ *B*_*k*_) acts like a 0 in the resulting space. Then the number of cavities of dimension *k* will be the dimension of *H*_*k*_, since *H*_*k*_ is generated by equivalence classes of *k*-cycles, with each of these nontrivial equivalence classes corresponding to a cavity within the simplicial complex. Indeed, by examining the simplicial complex shown in Figure 4D, we see that one cavity is enclosed by 1-simplices, and by direct calculation we can verify that dim *H*_1_ = 1 with the one non-trivial equivalence class represented by any one of the green cycles.

The sequence of linear maps in a chain complex gives rise to a sequence of homology groups that are the “compressed version” of the chain complex. The dimension of the *k*-th homology group counts the number of topological cavities enclosed by *k*-cycles (including *H*_0_, which counts the number of connected components). We show examples of cavities and the lack thereof in Figure 4E for dimensions 1 through 3. The dimension of the *k*-th homology group *β*_*k*_ = dim(*H*_*k*_) = dim(*Z*_*k*_) − dim(*B*_*k*_) is called the *k*-th Betti number. Betti numbers are topological invariants of the simplicial complex and are related to the well-known Euler characteristic via *χ* = $\sum_{i=0}^{\infty }$(−1)^*i*^ (# of *k*-simplices) = $\sum_{i=0}^{\infty }$(−1)^*i*^ *β*_*i*_.

## HOMOLOGY FROM COMPLEX TO COMPLEX: PERSISTENT HOMOLOGY

When considering data analysis, we have thus far discussed binary simplicial complexes such as the clique complex of a binary graph. However, biological relations often have intensities manifesting as weights on these relations (e.g., streamline counts between brain regions, functional similarity between neuronal activity time series, co-expression of genes, etc.). Answering the question of how to optimally (or even adequately) incorporate this additional information into our representations of the system and associated analyses remains a challenge. One approach for analyses of weighted graphs is to choose a threshold on the edge weight, and to retain only edges above this threshold. However, this approach requires that one make a very strict decision about which edges are relevant and which edges are not. Here we will circumvent this choice by thresholding the weighted graph at all values to obtain a sequence of binary simplicial complexes. As a result, we will be able to follow cavities throughout the simplicial complex sequence.

### Filtrations

Imagine flipping through a *z*-stack or a series of two-dimensional images of a cell from the top to the bottom. You might mentally note the positions of organelles, membranes, etc., in slice *i* to encode how they map into slice *i* + 1. We are going to use this idea to “flip through” slices of a weighted graph.

**Figure 5.**
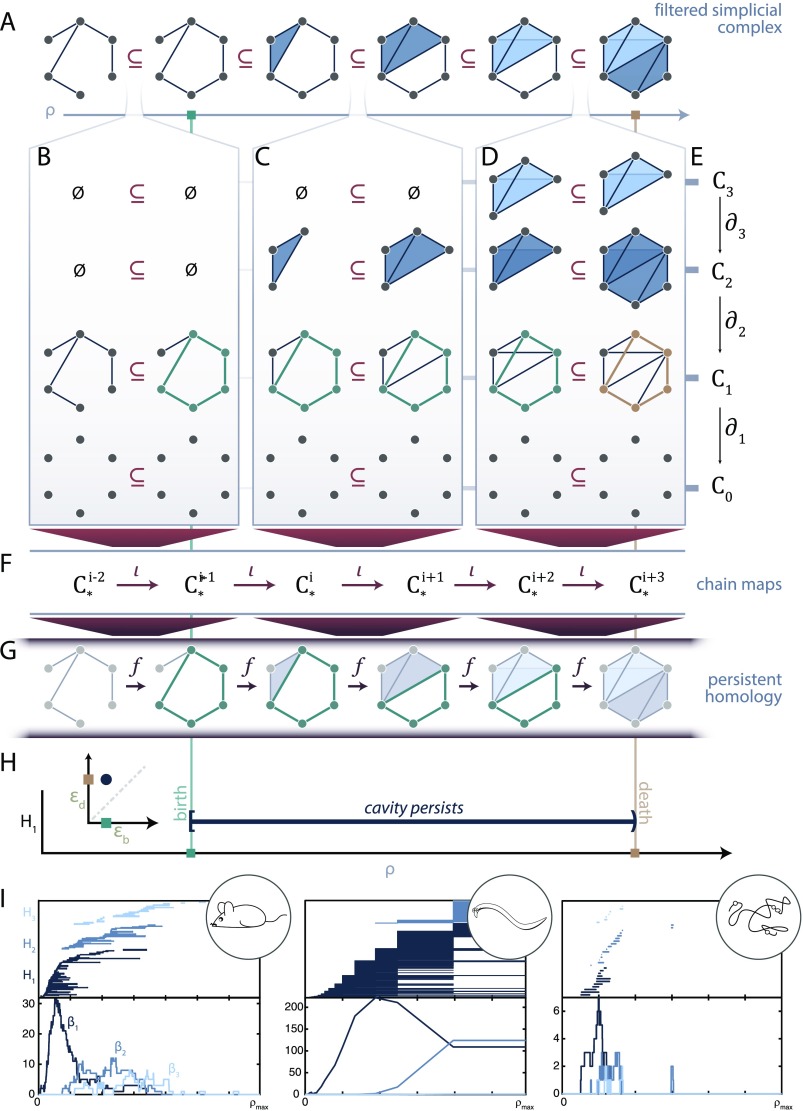
Persistent homology. (A) Filtered simplicial complex along the parameter *ρ*. (B, C, D) Considering the filtration along each dimension. A persistent cycle (green) is born in panel (B), persists through panel (C), and dies in panel (D). Each simplicial complex has an associated chain complex (E). Then we get *ι* that map chain complexes to chain complexes (F). Finally these maps induce maps *f* between homology groups (G). (H) We can record the birth and death time of this example persistent cycle (green cycle as representative (G)) as a barcode or persistence diagram (inset). (H) Barcodes (top) and Betti curves (bottom) for the mouse connectome (left), *C. elegans* electrical and chemical synapses (middle), and genomic interaction data (right).

In practice, we often begin with a model of a system as an edge-weighted graph, from which we can simply derive an ordering of the edges from greatest to least. We can then add edges to the empty graph following this ordering, resulting in a sequence of binary graphs where each graph in the sequence is a subgraph of the next graph in the sequence. What we have just described is an example of a filtration, or a sequence of objects *G*^0^, *G*^1^, … with each *G*^*i*^ ⊆ *G*^*i*+1^. From each binary graph we can construct the clique complex (see Box 1), which finally induces the desired filtration of simplicial complexes. Although creating a filtration from a weighted graph in this way is common (called the order complex; Giusti et al., 2015; A. Sizemore et al., 2017; or weight rank clique filtration; Petri et al., 2014, 2013), all we really need is a weighted simplicial complex in which weights |.| on each simplex follow the rule *s*′ ≤ *s* ⇒ |*s*′| ≤ |*s*| (for an example with weights on nodes rather than edges, see A. E. Sizemore, Karuza, Giusti, & Bassett, 2018). Then we can create a sequence of simplicial complexes by adding simplices one at a time in order of decreasing weight, which we indicate by the parameter *ρ* in Figure 5A.

Creating a filtered simplicial complex from a weighted simplicial complex—such as that constructed from a weighted graph—is quite useful. It relieves the burden of finding analysis techniques that extend to weighted simplices, and it also preserves the relational information contained in the original weights by encoding such information in the ordering. This dependence on the order of weights, rather than on the numerical value of the weights, can prove particularly useful in the study of empirical recordings in which the raw measurement values are not fully reliable. It is also of course possible to map results back to the weights in experiments where the originally measured empirical values are critical.

Now we have a filtered simplicial complex with complex *K*^0^ ⊆ ⋯ ⊆ *K*^*T*^, and we know how to map simplices from the *i*^*th*^ complex *K*^*i*^ into the next complex *K*^*i*+1^: we send each simplex *s* ∈ *K*^*i*^ to its natural counterpart in *K*^*i*+1^ (Figure 5A). We break down this inclusion by dimension in Figure 5B–D. Recall that from each simplicial complex along the filtration, we can create a chain complex (Figure 5E). Then if we can send *k*-simplex *s* in *K*^*i*^ to the *k*-simplex *f*(*s*) ∈ *K*^*i*+1^, we also immediately get maps from *C*_*k*_(*K*^*i*^) to *C*_*k*_(*K*^*i*+1^) because we know how to map the basis elements: they correspond to simplices. These are called chain maps *ι* : *C*_*k*_(*K*^*i*^) → *C*_*k*_(*K*^*i*+1^) (Figure 5F), and they are defined from our knowledge of *K*^*i*^ ⊆ *K*^*i*+1^. Thus, we can nicely map simplices to simplices, and paths to paths (see Figure 5B–D), and we can similarly map *k*-chains to *k*-chains across the filtration (Figure 5F).

### Persistent Homology

Although the mathematics that we have thus far discussed are interesting in and of themselves, the true impact of topological data analysis appears when passing from a single complex to an evolving filtration. Note in Figure 5E that there is a reminder about the chain complex with boundary maps from *C*_*k*_(*K*^*i*^) → *C*_*k*−1_(*K*^*i*^). Using Figure 5D as a visual example, we note that if we map an element from the 2-skeleton across to the 2-skeleton of the next complex and then down to the 1-skeleton, we get the same result as when we instead map first down to the 1-skeleton and then across to the next complex. When we move to chain maps, although less easy to directly visualize, the same property holds; that is, going across and then down is the same as going down and then across. If we think of chain complexes as pieces with assembly rules, then performing the construction and then moving to the next chain complex is the same as moving to the next chain complex and following these new, but compatible, assembly instructions.

How does this process relate to topological compression? Say that we have two equivalent 1-cycles ℓ_1_ ∼ ℓ_2_ ∈ *C*_1_(*K*^*i*^). Then we know that they must still be equivalent in *C*_1_(*K*^*i*+1^) since boundaries map to boundaries and cycles to cycles and thus we can map equivalence classes of cycles from one complex to the next. In moving from one complex to the next, we might form a new cycle (Figure 5B, green), map a nontrivial cycle to a nontrivial cycle (Figure 5C, green), or map a nontrivial cycle to a boundary cycle (Figure 5D, green to gold). From our chain maps we get induced maps on the homology groups *f*_*k*_ : *H*_*k*_(*K*^*i*^) → *H*_*k*_(*K*^*i*+1^) (visualized in Figure 5G; note that we have suppressed notation and write *H*_*k*_(*K*^*i*^) to mean *H*_*k*_(*C*_*k*_(*K*^*i*^)) for simplicity). Many equivalence classes can survive these mappings, and together this is the persistent homology of the filtered simplicial complex.

Having maps between homology groups means that we can identify the point along the filtration at which a cavity (i.e., a non-trivial equivalence class of *k*-cycles) is first formed (Figure 5B and G, green line). Then we can follow this particular cavity as we add more simplices (Figure 5C and G), and finally we can know the point at which this cavity is filled (Figure 5D and G; the non-trivial equivalence class maps to the trivial equivalence class of boundary cycles). It is important to note that we highlight in our illustration only one representative *k*-cycle in each equivalence class to represent the persistent cavity, although we could have chosen any equivalent cycle as a representative. The first appearance of a persistent cavity is called its birth, the value at which it is filled is known as its death, and the difference between the death and birth is called the lifetime of the persistent cavity. This process assigns a half-open interval (*b*, *d*) to the persistent cavity with *b*, *d* the values of the persistent cavity birth and death, respectively. Then all persistent cavities within a filtered simplicial complex can be visualized as part of a barcode (Figure 5H) or a point (*b*, *d*) on the extended half-plane (persistent cavities that never die are given a death value of ∞) called the persistence diagram of dimension *k* (Figure 5H, inset).

The barcode in each dimension defines a signature of the filtered simplicial complex that describes how topological features evolve along the filtration, providing insight into local-to-global organization. Importantly, a barcode gives more than a summary statistic; it shows not only the existence or number of persistent features, but also when they arise and overlap along the filtered simplicial complex. For example, the filtered clique complex of a weighted ring graph would produce one long-lived 1-cycle, arising from the one circular loop. Since a long-persisting cavity avoids death for an extended period of time, we often assume that long-lived persistent cavities describe more fundamental features of the complex, while short-lived cavities may be the natural consequence of noise in the system (although this is not always the case; see Kanari et al., 2018; B. J. Stolz, Harrington, & Porter, 2017). Previous work has used these persistence intervals to study and classify weighted networks (Horak et al., 2009; Petri et al., 2013; A. Sizemore et al., 2017) and to identify topological changes in cerebral arteries that track with a participant’s age (Bendich, Marron, Miller, Pieloch, & Skwerer, 2016).

Here we compute the persistent homology of the mouse connectome weighted by streamline count, the *C. elegans* electrical and chemical synapse circuit weighted by number of connections, and genomic Hi-C data weighted by physical interaction frequency (Figure 5I). We plot the barcode (above) and Betti curves *β*_*k*_(*ρ*) = *k*th Betti number at filtration parameter *ρ*. In Figure 5I we see that the mouse interareal connectome has a few long-lived 1-cavities and little higher dimensional persistent homology, similar to the persistent homology previously reported for the structural connectome of humans (A. E. Sizemore et al., 2017). In comparison, the persistent homology from the *C. elegans* electrical and chemical synapses shows many more persistent cavities in addition to those of higher dimensions. Finally, the persistent homology of the genomic interaction data reveals only a few persistent cavities, which is expected because of the high number and degrees of simplices (Figure 2C) if we assume this system is well modeled by the random geometric complex (M. Kahle, 2011). Although all three datasets are embedded in ℝ^3^, we speculate that higher dimensional features may still play an important role in the system’s function, such as in the computational capacity of the two neuronal networks.

Once the persistent homology has been computed, we can compare barcodes or persistence diagrams using a few different methods. First, we note that these objects are well studied even from a theoretical point of view for simplicial complex models known as the random-weighted graph (or random clique complex; Kahle, 2009) and the random geometric complex (Bobrowski, Kahle, & Skraba, 2015; Kahle, 2011). Thus, by visually comparing the persistence diagrams of the empirically observed structure to that expected in these well-studied models, one can deduce that the system may not be totally random or geometric (Giusti et al., 2015). Others have compared the persistence diagrams of weighted network models by using cavity lifetimes, weighted integrals of Betti curves, birth times, and more (Horak et al., 2009; Petri et al., 2013; A. Sizemore et al., 2017). One can also compute the *bottleneck distance* between persistence diagrams (Cohen-Steiner, Edelsbrunner, & Harer, 2007) or the distance between a slightly modified version of the barcode called a persistence landscape, which has some nicer statistical properties (Berry, Chen, Cisewski-Kehe, & Fasy, 2018; Bubenik, 2015; B. J. Stolz et al., 2017).

### Extracting Topological Features

Since each bar in the barcode arises from a particular persistent topological feature, one might hope to extract information about the “most important” or “most robust” topological features observed. This goal is, in fact, a more complicated problem than might be obvious at first glance, but nevertheless a solvable and tunable one.

**Figure 6.**
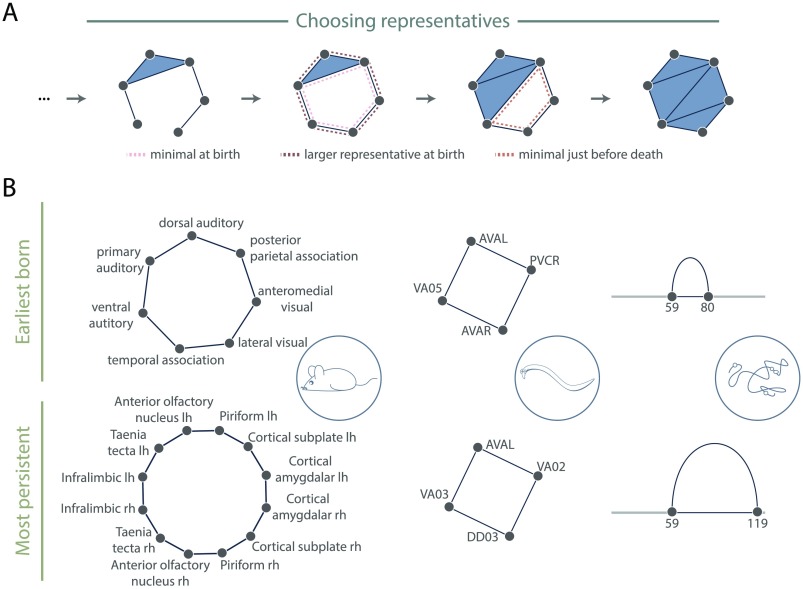
Options for extracting generators. (A) Filtered simplicial complex with one persistent homology class of dimension 1. As examples of possible representatives the minimal generator at birth (pink dashed), a larger cycle at birth (brown dashed), and the minimal midlife or just before death (orange dashed) are highlighted. (B) (Top) A representative at the birth time of the earliest born and (bottom) longest lived persistent cavities in the mouse interareal connectome (left), *C. elegans* electrical and chemical synapses (middle), and genomic Hi-C interactions (right) with the gray line representing linear genomic position, and with the blue loop representing the nodes and edges involved in the representative cycle.

First, if we only wish to determine the nodes and simplicies that surround a cavity—how difficult could that be? Recall that in persistent homology we are mapping *equivalence classes* corresponding to cavities through the filtration. This means that to extract the loop, we must *choose* a particular representative (or set of representatives) from the equivalence class of a cavity-surrounding loop. Often the representative(s) with minimal hop distance (number of edges) at the birth index is(are) used (see Figure 6A, pink dashed line) since biological systems often utilize shortest paths. We note that there may exist multiple representatives with minimal hop distance (e.g., attach a fifth node to three of four nodes in a diamond and fill in all triangles as 2-simplices), and thus care must be taken if the later analyses and interpretations require only one representative cycle per persistent homology class. For reasons related to the system under study, one may be most interested in minimal cycles at just before the death time (Figure 6A, orange dashed line), or some large cycle in the middle of the persistent cavity lifetime. Still, the minimal generators may not always be the most relevant, and we emphasize that no one strategy exists for choosing generators—it instead should be determined by the system and question at hand. Many persistent homology software implementations will report a representative cycle for each persistent homology class, although often this is used for computing the barcodes only and there are no guarantees that the cycle that is identified is geometrically nice (Adams & Tausz, 2011; Henselman & Ghrist, 2016). However, methods now exist to find the generator optimizing some other parameter, for example, a given weighting on the simplices (Dey, Hirani, & Krishnamoorthy, 2011), persistence (Busaryev, Dey, & Wang, 2010), or minimal length along the genome (Emmett, Schweinhart, & Rabadan, 2016). No one method may be ideal for all analyses, and instead the choice of method represents an area of optimization tunable to the system under study. Future work may expand this set of methods through the development of additional novel algorithms.

The second part of this question is how to determine if a persistent cycle is significant. As noted above, we generally consider the longest lived persistent cycles to be the most intrinsic to the filtered simplicial complex. Additionally, we may find cavities appearing much earlier in the filtration than expected given some null model (Bobrowski et al., 2015; Kahle et al., 2013; A. E. Sizemore et al., 2017; B. J. Stolz et al., 2017). To illustrate these ideas, we show in Figure 6B a representative cycle at birth from the earliest born (top) and longest-lived (bottom) persistent cavities in our datasets. For the sake of the pedagogical nature of this tutorial, we keep our calculations simple and report cycles returned by the Eirene software (Henselman & Ghrist, 2016). For the structural brain network, one might argue that the earliest born cavities are the most crucial since they involve the highest weighted edges of the system. For genomic interaction data, in contrast, at this particular scale the most persistent cycle could be more interesting since it can indicate larger long-distance interactions (Emmett et al., 2016).

## CONCLUSION

The language of algebraic topology offers powerful tools for network scientists. Using three example datasets, we illustrated how to translate a network into a simplicial complex and both apply and understand persistent homology, concluding with results and reasonable interpretations. Now, for the interested network neuroscientist, we revisit our initially posed questions, hoping that our deeper understanding of the subject will spur the generation of new ideas. What does a cavity mean in any given scientist’s system of interest? How would one define small groups making functional units? As with any method it is important to understand both the fundamental assumptions and underlying mathematics.

Moreover, we can push even further than the methods demonstrated in this paper. Applied topology is only about two decades old, but has nevertheless captured the attention of scientists and mathematicians alike. We have since charged past the initial applications and can now use topology to answer more complicated and detailed questions. Does the system of interest have weighted nodes instead of weighted edges (A. E. Sizemore et al., 2018)? Is the system time varying with either unweighted (Botnan & Lesnick, 2016; Carlsson & De Silva, 2010) or weighted edges (Cohen-Steiner, Edelsbrunner, & Morozov, 2006; Munch, 2013; Yoo, Kim, Ahn, & Ye, 2016)? Would one perhaps wish to obtain circular coordinates for the data (De Silva, Morozov, & Vejdemo-Johansson, 2011; Rybakken, Baas, & Dunn, 2017)? Would equivalence classes of paths instead of cycles be useful (Chowdhury & Mémoli, 2018)? Does one need distances between networks (Chowdhury & Mémoli, 2015) or multiple filtering parameters (Carlsson, Singh, & Zomorodian, 2009; Lesnick & Wright, 2015)? These and more questions are recently answerable using concepts developed in applied topology.

To conclude, the translation of data into the language of algebraic topology opens many doors for analysis and subsequent insight. Those scientists with an understanding of this language can add a myriad of powerful tools to their analysis repository. Additionally, continued discussion between the mathematicians developing the tools and the scientists applying the tools will continue to spur methodical advances with biological questions as the driving force. We hope from these local interdisciplinary collaborations that a larger, global understanding of neural systems will emerge.

## ACKNOWLEDGMENTS

The authors would like to thank Richard Betzel, Harvey Huang, and Sunnia Chen for helpful discussions.

## AUTHOR CONTRIBUTIONS

Ann Sizemore: Formal analysis; Investigation; Software; Visualization; Writing – original draft; Writing – review & editing. Jennifer Phillips-Cremins: Writing – review & editing. Robert Ghrist: Writing – review & editing. Danielle Bassett: Conceptualization; Funding acquisition; Project administration; Supervision; Writing – review & editing.

## FUNDING INFORMATION

Danielle Bassett, John D. and Catherine T. MacArthur Foundation (US). Danielle Bassett, Alfred P. Sloan Foundation (http://doi.org/10.13039/100000879). Danielle Bassett, Paul G. Allen Family Foundation (US). Danielle Bassett, Army Research Laboratory (US), Award ID: W911NF-10-2-0022. Danielle Bassett, Army Research Office (US), Award ID: Bassett-W911NF-14-1-0679, Grafton-W911NF-16-1-0474, DCIST-W911NF-17-2-0181. Danielle Bassett, Office of Naval Research (http://doi.org/10.13039/100000006). Danielle Bassett, National Institute of Mental Health (http://doi.org/10.13039/100000025), Award ID: 2-R01-DC-009209-11, R01 – MH112847, R01-MH107235, R21-M MH-106799. Danielle Bassett, National Institute of Child Health and Human Development (http://doi.org/10.13039/100000071), Award ID: 1R01HD086888-01. Danielle Bassett, National Institute of Neurological Disorders and Stroke (http://doi.org/10.13039/100000065), Award ID: R01 NS099348. Danielle Bassett, National Science Foundation (http://doi.org/10.13039/100000001), Award ID: BCS-1441502, BCS-1430087, NSF PHY-1554488 and BCS-1631550. Danielle Bassett, ISI Foundation. Danielle Bassett, National Science Foundation and National Institute of General Medical Sciences, Award ID: 1562665. Robert Ghrist, Office of Naval Research (https://doi.org/10.13039/100000006), Award ID: N00014-16-1-2010.

## TECHNICAL TERMS

Persistent homology: The collection of persistent cavities across a filtration of simplicial complexes.
*k*-Simplex: *k* + 1 nodes and their convex hull, assuming all nodes are affinely positioned.
Boundary operator: Linear map sending a chain to its boundary.
*k*^*th*^ Chain group: the vector space with basis elements corresponding to *k*-simplices in a simplicial complex.
k-cycle subspace: Vector space with k-cycles as basis elements.
*k*-Boundary subspace: Vector space whose basis elements are those k-chains that correspond to the boundary of a (k + 1)-simplex.
Equivalence class: Set of elements that are all equivalent to each other.
*k*^*th*^ Homology group: Vector space in which each basis element is a non-trivial equivalence class of k-cycles.
Filtration: Sequence of objects in which each object is a subobject of the next.
Representative cycle: One particular cycle extracted from an equivalence class of cycles.
